# Assessment of angle-dependent spectral distortion to develop accurate hyperspectral endoscopy

**DOI:** 10.1038/s41598-022-16232-0

**Published:** 2022-07-13

**Authors:** Jungwoo Lee, Jonghee Yoon

**Affiliations:** Department of Physics, Ajou University, Suwon, Republic of Korea

**Keywords:** Applied optics, Optics and photonics, Biophotonics

## Abstract

Hyperspectral endoscopy has shown its potential to improve disease diagnosis in gastrointestinal tracts. Recent approaches in developing hyperspectral endoscopy are mainly focusing on enhancing image speed and quality of spectral information under a clinical environment, but there are many issues in obtaining consistent spectral information due to complicated imaging conditions, including imaging angle, non-uniform illumination, working distance, and low reflected signal. We quantitatively investigated the effect of imaging angle on the distortion of spectral information by exploiting a bifurcated fiber, spectrometer, and tissue-mimicking phantom. Spectral distortion becomes severe as increasing the angle of the imaging fiber or shortening camera exposure time for fast image acquisition. Moreover, spectral ranges from 450 to 550 nm are more susceptible to the angle-dependent spectral distortion than longer spectral ranges. Therefore, imaging angles close to normal and longer target spectral ranges with enough detector exposure time could minimize spectral distortion in hyperspectral endoscopy. These findings will help implement clinical HSI endoscopy for the robust and accurate measurement of spectral information from patients in vivo.

## Introduction

Recently, hyperspectral imaging (HSI) has shown potential as a disease diagnostic tool and surgical guidance tool in clinics^1,2^. HSI measures both spatial and spectral information that could provide tissue morphological and biochemical features without exogenous labeling agents, enhancing the contrast between healthy tissue and lesions by harnessing advanced image processing methods or artificial intelligence to analyze hyperspectral images with rich information^1^. Thus HSI enables the improved disease diagnosis compared to conventional color imaging methods due to indistinguishable color differences in lesions^3^. There were many reports that HSI is a versatile tool for examining biopsy samples^4,5^ or exposure tissue during open surgery^6,7^.

Moreover, hyperspectral endoscopy has been implemented to measure HSI from the inside patient in vivo using flexible optical fiber bundle and spectral imaging methods such as line-scanning^8^ and snapshot methods^9^. Previous reports demonstrated that the spectral imaging endoscope could capture significant spectral information from patients, discriminating between healthy tissues and lesions^8–10^. Although hyperspectral endoscopy has shown its potential as a disease diagnostic tool, measuring consistent spectral information from patients in vivo remains challenging due to uncontrolled imaging conditions inside the body, including imaging angle^11^, working distance^12^, fiber diameter^13,14^, and spontaneous patient movements^8,15^.

**Figure 1:**
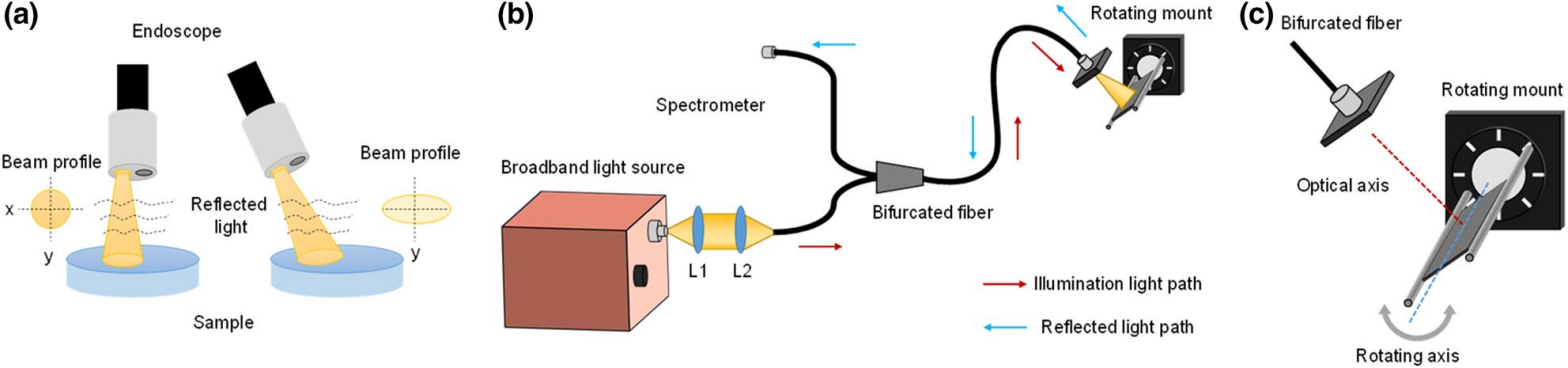
(**a**) schematic diagram of endoscopic imaging with normal and tilted positions, respectively. (**b**) An optical system for measuring angle-dependant hyperspectral imaging via exploiting a bifurcated fiber. A high-power broadband light source was coupled to the bifurcated fiber, and reflected light from a sample was collected and delivered to a spectrometer. (**c**) The angle of the fiber was quantitatively controlled by using a rotating mount. Hyperspectral signals were measured at varying angles from normal to 60 degrees.

These uncontrolled optical parameters make it difficult to quantify accurate reflectance and absorbance of tissue which can be calculated by dividing spectral profiles of measured samples with the spectral profiles of illumination light. Our previous work found that illumination power varies depending on the working distance of the endoscope, which cannot be quantitatively controlled inside the body^12,16^. To overcome this issue, the methods for correcting non-uniform illumination were developed for estimating illumination power using near-infrared light^12^. It has shown the capability of estimating accurate spectral features independent of working distances. However, we found that there were spectral distortions unable to be corrected using the non-uniform illumination correcting methods. Supplementary figure 1 shows spectral profiles of blood measured by a clinical hyperspectral endoscope at three different imaging conditions, and there are severe spectral distortions at the spectral range below 550 nm. A previous study reported that there is angle dependency in fluorescent imaging^17^, which indicates that measuring spectral information is also affected by the imaging angle of the endoscope. To study the consistency of spectral information under in vivo endoscopic imaging conditions, HSI was performed at various illumination and image acquisition conditions (Fig. 1a).

Here, we investigated the effects of optical parameters such as imaging angle and speed on the quality of spectral information measured using an optical fiber. The spectral distortions were assessed by adjusting the fiber angle and detector exposure time. In addition, the effects of scattering properties of the sample on spectral imaging were also studied by using tissue-mimicking phantoms with various scattering properties. Spectral distortion becomes severe as increasing imaging angles or shortening exposure time. Moreover, the scattering properties of biological materials seem not to affect the consistency of spectral imaging significantly. These findings will help implement clinical HSI endoscopy for the robust and accurate measurement of spectral information from patients in vivo.

## Methods

### Spectral imaging system

In order to test the effect of endoscopic imaging angles on hyperspectral signal distortions, a hyperspectral fiber imaging system with a rotating sample mount was established (Fig. 1b). A bifurcated fiber (BFY105, Thorlabs) was exploited to illuminate the sample and measure reflected light at the same angle, which is consistent with imaging conditions with hyperspectral endoscopy. A high-power broadband light source (OSL2, Thorlabs) was coupled to one end of the bifurcated fiber using lenses. Illumination light was delivered to the sample, and reflected light was collected via the bifurcated fiber and delivered to a spectrometer (ULS2048CL-EVO, Avantes) to measure a spectral profile of the reflected light. The rotating sample mount was developed using a manual rotating mount (CRM1T, Thorlabs). The angle of the rotating mount was initially set to a 0-degree angle, and the bifurcated fiber was aligned at the normal position. Then imaging angles were quantitatively adjusted via exploiting the rotating sample mount from normal (0-degree angle) to 60 degrees (Fig. 1c).

### Spectral signal normalization

In order to quantify the true reflectance of the target, the spectral normalization process of the measured spectral information was performed by dividing the spectral profiles of a target by the reference spectral profile of the light source using a white target. The white target reflected the illuminating light with negligible absorption; thus, the hyperspectral signal obtained from the white target can be used as the spectral profile of the light source. The normalized reflectance can be calculated via the following Eq. 1:

1 $$ R\left( \lambda \right) = \frac{{I\left( \lambda \right) - I_{dark} }}{{I_{0} \left( \lambda \right) - I_{dark} }}, $$

where R(λ) is the normalized reflectance at a given wavelength (λ). I, I_0_, and I_dark_ are the intensities of the spectral signals measured from the sample and white reflectance (paper) and the dark signal obtained without light illumination, respectively.

### Spectral angle mapping

For the quantitative analysis of the consistency of measured spectral signals at various imaging conditions, spectral angle mapping (SAM) was exploited^18^. SAM quantifies the similarity between two spectral signals (target and reference spectral signals) by calculating the angles, α, using the following Eq. 2:

2 $$ \alpha = cos^{ - 1} \left( {\frac{{\mathop \sum \nolimits_{\lambda = 1}^{n} t_{\lambda } r_{\lambda } }}{{\left( {\mathop \sum \nolimits_{\lambda = 1}^{n} t_{\lambda }^{2} } \right)^{0.5} \left( {\mathop \sum \nolimits_{\lambda = 1}^{n} r_{\lambda }^{2} } \right)^{0.5} }}} \right), $$

where t_λ_ and r_λ_ are normalized reflectance values of the target and reference at a given wavelength(λ), respectively. *n* indicates the total number of spectral channels. If two spectral signals are similar, then the α is close to 0; otherwise, the α has a large value.

### Tissue-mimicking phantom

The effects of the angle of the fiber on the accuracy of the spectral measurement were tested via exploiting a tissue-mimicking phantom. Tissue-mimicking phantom was fabricated using agar and intralipid to control the scattering property. The protocol was described in the previous report^19^. All chemicals were purchased from Sigma-Aldrich. Briefly, 0.75 g of agar was dissolved in boiled water. Once the temperature of the agar solution reached around 30–40 °C, then intralipid was added to the solution with vortexing. The mixture was poured into a sample holder and put for 2–3 h to gel the agar solution.

A previous report showed that the scattering coefficients were linearly increased according to intralipid concentration^20^. Thus four tissue-mimicking phantoms were prepared with different intralipid concentrations (2.08%, 4.16%, 6.24%, and 8.32%) to investigate the effects of the scattering property and angle of the fiber on the measurement of spectral profiles. Four tissue phantoms have reduced scattering coefficients of 0.05 cm^−1^, 0.10 cm^−1^, 0.15 cm^−1^, 0.20 cm^−1^, respectively^19^. These reduced scattering coefficient ranges were determined to follow generic optical properties of biological tissue^21^.

### Software

Matlab R2020b was used for the acquisition of spectral information using the spectrometer the quantitative data analysis.

## Results

### Large imaging angle occurs spectral distortion

First, the effect of imaging angle on the quality of HSI was investigated. A bifurcated fiber was exploited to perform HSI under similar conditions to clinical endoscopy, which illuminates a tissue and collects reflected light signal using the same optical fiber. A broadband light source with spectral ranges from 400 to 850 nm was coupled to the one end of the bifurcated fiber, and a spectrometer was connected to the other end of the fiber to measure spectral information of the reflected light from the sample. The fiber angle was quantitatively adjusted from 0° (normal) to 60° via the rotating mount. A piece of white paper, a highly scattering material with negligible absorbance in visible light, was used as a reflectance target to measure the spectral profile of the illumination light.

**Figure 2:**
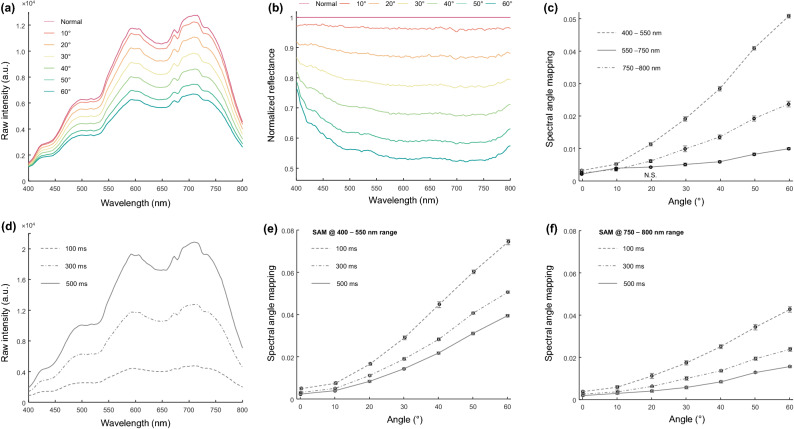
Quantitative analysis of spectral profiles of the light source measured at various angles. (**a**) Raw spectral profiles of the light source were measured via the spectrometer with an exposure time of 300 ms. (**b**) Spectral profiles were normalized by exploiting the spectral signal obtained at the normal angle as a reference signal. (**c**) SAM results of three spectral regions, 400–550 nm (dot-dash line), 550–750 nm (solid line), and 750–800 nm (dashed line). (**d**) Average reflected signals of the light source measured at the normal position with an exposure time of 100 ms (dashed line), 300 ms (dot-dash line), and 500 ms (solid line). (**e**, **f**) SAM results of 400–550 nm and 750–800 nm, respectively.

Figure 2a shows the spectral profiles of reflected light from the white paper measured at various imaging angles. The angle of reflection increases as imaging angles change from normal to 60°; thus, the amount of light received by the fiber is diminished. Although measured intensity at the fiber is decreased, the spectral profiles should remain the same due to the negligible absorbance of the paper at the visible wavelength. To check whether the imaging angle affects the measured spectral profile, normalized reflectance was calculated by using the spectral profile measured at the normal angle as the reference (See “Methods” section). Figure 2 shows normalized reflectance obtained at various fiber angles. As increasing the fiber's angle, spectral profiles show nonuniform reflectance over the wavelength. Specifically, spectral profiles at 400–550 nm and 750–800 nm show increased reflectance at large fiber angles, while there was relatively stable reflectance at the spectral range of 550–800 nm. Therefore the three ranges (400–550 nm, 550–750 nm, and 750–800 nm) were selected for investing the effect of imaging angles on spectral distortion at each spectral range.

To quantify this nonuniformity of spectral profiles of reflectance, spectral profiles were segmented in three ranges: 400–550 nm, 550–750 nm, and 750–800 nm, and the segmented spectral signals were quantitatively analyzed via SAM. Figure 2c shows SAM results of three spectral ranges over the fiber angles. All SAM values are increased as increasing the angle of the fiber. But there were huge differences in the spectral ranges at 400–550 nm and 750–800 nm compared to the SAM result calculated from the spectral range at 550–750 nm. Moreover, spectral distortion in 400–550 nm is much larger than in 750–800 nm.

### Low reflected signal and fast imaging speed affect the quality of HSI

We found that the light source has low power in the spectral ranges that showed severe spectral distortion in the previous result. Thus, the effect of low reflected signals on the spectral distortion was assessed by changing the exposure time of the spectrometer under the same illumination conditions. Three exposure time (100, 300, and 500 ms) were selected because measured spectral signals clearly showed low, intermediate, and high intensities enough to investigate whether the measured intensity level at the detector affects spectral distortion. Figure 2d shows the three reflected spectral profiles measured at the normal angle with exposure times of 100, 300, and 500 ms, respectively. Raw intensities were increased as increasing the exposure time of the spectrometer. Figure 2e, f show the SAM results in 400–550 nm and 750–800 nm calculated using the spectral profile measured at the normal angle at each exposure time as reference signals. There were significant spectral distortions at low exposure time, which means detector noise and low exposure time affect the accuracy of spectral measurement. In addition, spectral distortions in 400–550 nm are larger than those in 750–800 nm, consistent with the previous result.

### Scattering properties of biological tissue have little effect on HSI

**Figure 3:**
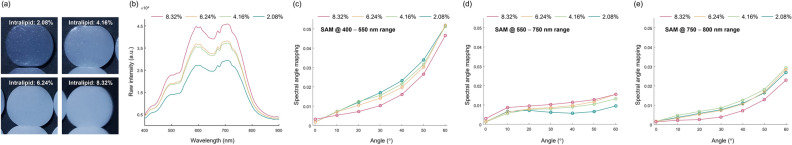
Assessment of angle-dependent spectral distortion by exploiting tissue-mimicking phantoms. (**a**) Photographs of a tissue-mimicking phantom with intralipid concentrations of 2.08%, 4.16%, 6.24%, and 8.32%. (**b**) Raw reflected signals from phantoms were measured at the normal position. Reflected signals showed high intensity as increasing intralipid concentrations. (**c**–**e**) SAM results of four tissue-mimicking phantoms were quantitatively analyzed at three different spectral regions (400–550 nm, 550–750 nm, and 750–800 nm), respectively.

Previous results indicate more spectral distortions in a shorter wavelength than a longer wavelength. We test whether the wavelength dependency in the spectral distortion is related to the scattering properties of the target. Thus, the effects of light scattering were assessed by using tissue-mimicking phantoms. Tissue-mimicking phantom was constructed using agarose with various intralipid concentrations to control scattering coefficients of the phantoms (See “Methods” section). Four tissue-mimicking phantoms with intralipid concentrations of 2.08%, 4.16%, 6.24%, and 8.32% were measured with the exposure time of 300 ms, respectively. The maximal exposure time was chosen at which the measured spectral signal from tissue phantoms did not saturate. As increasing intralipid concentration, the sample became white due to increased light scattering (Fig. 3a). Therefore, there are more backscattered light signals from the phantoms with high intralipid concentration (Fig. 3b).

Figures 3c–e show the quantitative SAM analysis of spectral distortion at various fiber angles in the range of 400–550 nm, 550–750 nm, and 750–800 nm, respectively. Spectral distortions were increased as the fiber's angle increased. Moreover, there was a huge spectral distortion in the spectral range of 400–550 nm. These results are consistent with the results obtained from the white paper. On the other hand, there is no significant difference in spectral distortion among the samples with various intralipid concentrations. This result indicates that the effect of light scattering of biological tissues on hyperspectral imaging is less than that of the imaging angle.

In addition, spectral distortion was assessed using a large tissue-mimicking phantom as there was an increased illumination area at a large fiber angle beyond the sample boundary (Supplementary fig. 1a), which might cause errors in the quantitative analysis of spectral. Illumination areas were mainly placed on the large phantom regardless of fiber angles. Due to the sample size, spectral distortion was analyzed at three angles (normal, 10°, and 20°) at a long working distance. As the imaging conditions, including working distance, sample thickness, and diameter, were changed, the SAM values were larger than those measured in previous experiments. Although the quantitative results show a difference, the overall tendency of the angle-dependant spectral distortion at three spectral ranges is consistent with previous results.

## Discussion

The principle underlying hyperspectral image-based disease diagnosis is that there are differences in spectral profiles between healthy tissues and lesions; therefore, the accuracy of the measurement of spectral features is the most important in clinical applications of HSI. However, compared to ex vivo tissue imaging conditions, it is challenging to measure the high-quality spectral information under in vivo endoscopic imaging conditions due to flexible fiber with varying illumination and detection angles and working distance.

Here we assessed the effect of imaging conditions using the flexible fiber on the accuracy of hyperspectral imaging by adjusting a fiber angle and scattering property of tissue-mimicking phantoms. Quantitative analysis of a spectral profile using SAM shows increased spectral distortions as increasing imaging angles. Interestingly, spectral distortions in 400–500 nm and 750–800 nm are much larger than those measured in 550–750 nm. In addition, short exposure time decreases the accuracy of HSI due to detector noise. These experiments indicate that small backscattered signal or fast imaging conditions might limit the accuracy of HSI.

Effects of scattering properties of the sample were also analyzed by varying intralipid concentrations of the phantom. The quantitative analysis results show large spectral distortions in the range of 400–550 nm and 750–800 nm, consistent with the results obtained from the white paper sample. However, there are small differences in spectral distortions among various intralipid concentrations, which indicates that the fiber angle is a key parameter that affects the accuracy of HSI. And the scattering properties of biological tissue show a minimal effect on HSI.

We note that all experiments show the highest spectral distortions in the spectral range of 400–550 nm, which might be related to the nature of light scattering. A shorter wavelength of light in biological tissue is more scattered than a longer wavelength^22^. The shorter wavelength light occurs more scattering in the sample, and only a small portion of reflected light is measured at the detector, which might increase spectral distortion. This study concludes that the spectral property of a light source, exposure time, fiber angle, and target spectral ranges for the analysis are important parameters for clinical applications of HSI endoscopy. We found that an imaging angle of less than 20° and a long exposure time enough to get high average intensity could minimize spectral distortion in spectral information. When implementing HSI endoscopy and designing the protocol for HSI-based disease diagnosis and treatment, imaging conditions, light source, and a spectral window should be considered for accurate outcomes.

Based on the knowledge obtained from our previous experience of pilot clinical application of hyperspectral endoscopy, we found that it is extremely challenging to precisely control imaging conditions inside the patient. Maintaining the endoscope close to the target with an angle of less than 20° under in vivo imaging conditions requires a high-level endoscopic skill. One solution is to utilize a side-viewing endoscope to observe a luminal organ^23,24^, which helps control the distance between the endoscope and tissue and imaging angles. However, it also requires high-level skills of the endoscopist. Another solution is to develop a multimodal endoscopy system that combines a hyperspectral imaging method with other structural imaging techniques such as optical coherence tomography^25^, ultrasound imaging^26^, and photoacoustic imaging^26,27^. These methods provide 3D quantitative structural information which can be used to compensate for the uncontrolled imaging conditions to obtain the precise spectral signatures under in vivo patient imaging conditions. As this approach is an image-based correction method, it does not be dominated by the skills of the endoscopist.

In conclusion, obtaining accurate spectral features from the patient in vivo is the most important for the improved disease diagnosis using hyperspectral endoscopy. If the stable and precise hyperspectral image could be measured with well developed imaging protocols or combining other optical imaging methods, hyperspectral endoscopy would be a versatile in vivo disease diagnostic tool.

## Supplementary Information

Supplementary Figures.

## Data Availability

The datasets used and/or analysed during the current study available from the corresponding author on reasonable request.
