# Supplementary material for: Assessment of angle-dependent spectral distortion to develop accurate hyperspectral endoscopy
Source: Sci Rep. 2022 Jul 13;12:11892. doi: 10.1038/s41598-022-16232-0 (PMC9279473; doi:10.1038/s41598-022-16232-0)
Supplement: Supplementary file 1 — Supplementary Figures. [file 41598_2022_16232_MOESM1_ESM.pdf]

Supplementary Information for

Assessment of angle-dependent spectral distortion to develop accurate hyperspectral endoscopy

Lee et al.

This PDF file includes:

Supplementary Figures 1

Supplementary Figures 2

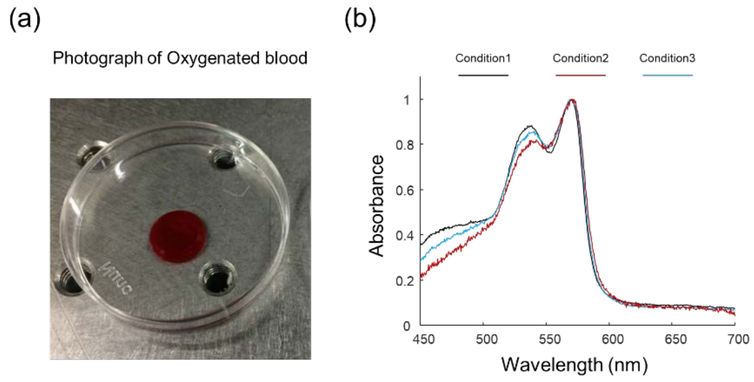

Supplementary Fig. 1 Spectral distortions measured from oxygenated blood using the hyperspectral endoscope. (a) Photograph of an oxygenated blood sample covered with a glass (b) Normalized absorbance of the blood sample measured at three different imaging conditions (working distance and angle).

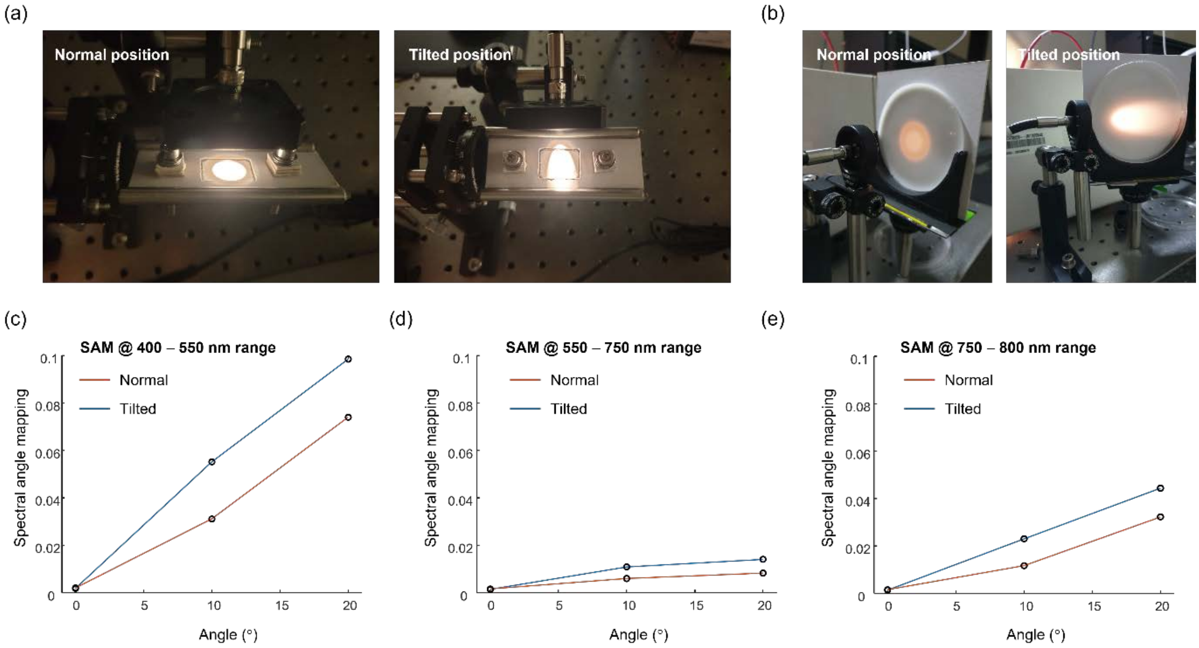

Supplementary Fig. 2 Spectral distortions at a large tissue-mimicking phantom. (a, b) Photographs of illuminating areas on small and large tissue-mimicking phantoms at normal and tilted positions, respectively. (c-e) SAM results from the large tissue-mimicking phantom with normal and tilted fiber positions were quantitatively analyzed at three different spectral regions (400 – 550 nm, 550 – 750 nm, and 750 – 800 nm), respectively.
